# Nonsuicidal Self-Injury and Suicide: The Role of Life Events in Clinical and Non-Clinical Populations of Adolescents

**DOI:** 10.3389/fpsyt.2020.00370

**Published:** 2020-05-06

**Authors:** Lili Olga Horváth, Dóra Győri, Dániel Komáromy, Gergely Mészáros, Dóra Szentiványi, Judit Balázs

**Affiliations:** ^1^Doctoral School of Psychology, Eötvös Loránd University, Budapest, Hungary; ^2^Institute of Psychology, Eötvös Loránd University, Budapest, Hungary; ^3^Faculty of Behavioural and Movement Sciences, Vrije Universiteit Amsterdam, Amsterdam, Netherlands; ^4^Faculty of Social and Behavioral Sciences, Universiteit van Amsterdam, Amsterdam, Netherlands; ^5^Mental Health Sciences, School of PhD Studies, Semmelweis University, Budapest, Hungary; ^6^Vadaskert Child Psychiatry Hospital and Outpatient Clinic, Budapest, Hungary; ^7^Pedagogical Assistance Services, Budapest, Hungary; ^8^Department of Psychology, Bjørknes University College, Oslo, Norway

**Keywords:** nonsuicidal self-injury, NSSI, suicidal behaviour, suicide, life events, adolescence

## Abstract

**Background:**

Nonsuicidal self-injury (NSSI) is highly prevalent in clinical and non-clinical populations of adolescents. Several studies have supported both the distinction and the strong association between NSSI and suicidal behavior. Although there is a great deal of data on the role of life events in both suicidal behavior and NSSI, few studies have assessed the role of life events in the NSSI–suicidal behavior relationship. Our aims were to explore the relationship between NSSI and suicidal behavior, and the possible moderating role of stressful life events in a clinical and non-clinical adolescent population.

**Method:**

A clinical (n = 202) and a nonclinical (n = 161) population of adolescents, aged 13–18 years were assessed. The Mini International Neuropsychiatric Interview Kid, Deliberate Self-Harm Inventory and the Life Events List were used. Group differences related to suicidal behavior, NSSI, and life events were tested with Wilcoxon tests. Two- and three-way interactions were tested with negative binomial regression models including zero-inflation parameter.

**Results:**

The prevalence of suicidal behavior (W = 7,306, p < .001), NSSI (W = 9,652, p < .001) and life events (W = 10,410 p < .001) were significantly higher in the clinical than in the non-clinical group. Between number of life events and NSSI, a moderate effect size (.38, 95%CI [.28,.46]) was found. The main effect of NSSI (*χ^2^*(1) = 109.65, p < .001) and group membership (*χ^2^*(1) = 39.13, p < .001) predicted suicidal behavior; the main effect of quantity of life events did not explain suicidal behavior. The interaction between NSSI and number of life events (*χ^2^*(1) = 10.49, p < .01) was associated with suicidal behavior. Among interpersonal, non-interpersonal events and adverse childhood circumstances, only interpersonal events were associated with both suicidal behavior (*χ^2^*(1) = 6.08, p < .05) and had a moderating effect (*χ^2^*(1) = 8.59, p < .01) on the NSSI–suicidal behavior relationship. Patterns of the effects of life events on the NSSI–suicidal behavior relationship did not differ in the two groups.

**Conclusion:**

Our results confirm the importance of prevention and intervention of NSSI, considering its high prevalence and frequent co-occurrence with suicidal behavior in both clinical and non-clinical adolescent populations. Moreover, to support NSSI and suicide prevention, we would like to highlight the importance of stressful life events, especially those associated with interpersonal conflicts, require special attention.

## Introduction

Nonsuicidal self-injury (NSSI) is defined as the direct and deliberate destruction of one’s own bodily tissue, with no observable intention to die as a consequence of the behavior, and for reasons not socially sanctioned (1, 2). The typical age of onset for NSSI is between 12 and 16 years (3, 4), and the behavior is highly prevalent in adolescence: lifetime prevalence is 15-46% in normal population (5–8) and as high as 40–80% in clinical populations (9). This alarmingly high prevalence implies NSSI is a major health issue not only because of the direct damage of the injuries themselves; recognition, but prevention and intervention of NSSI is also crucial because NSSI is associated with several internalizing and externalizing disorders (10), and is considered to be a strong predictor of suicidal behavior (11).

Although research supports the distinction between NSSI and suicidal behavior (12), and NSSI and suicide attempts typically differ in several key features—including intent, severity of medical damage, frequency (13) and number of methods (14)—the two phenomena are strongly associated: the overlap between NSSI and suicidal behavior is approximately 70% in clinical populations (14) and 50% in non-clinical populations (15). Possible pathways between NSSI and suicidal behavior have been described by several authors (16–18). One suggestion is based on the challenges of a clear-cut NSSI concept itself: as the nonsuicidal nature of NSSI can mostly be concluded from the person’s self-report, cases where the surface features of self-injury mask underlying suicidal intentions, or when the injury unintentionally leads to a lethal outcome, might be hard to categorize (19). Furthermore, a person can have ambivalent attitudes towards death during the self-injuring episode (1). Further theories include understanding NSSI as a “gateway” towards more severe forms of self-injury on a suicidal spectrum (16), or focus on self-injury as a process of habituation for fear and pain, thus making the person “capable” of suicide (17, 20). Moreover, shared risk factors (including shared psychiatric comorbidities and shared environmental risk factors, such as unsupportive family or high levels of stress) as third variables behind both NSSI and suicide (16, 17, 21) should also be taken into account, highlighting the role of interpersonal and broader environmental factors in the etiology and relationship of the two phenomena.

Broad theoretical and empirical evidence has previously suggested possible pathways between life events and both suicidal behavior (22, 23) and NSSI (24–26) separately. In line with the stress-diathesis models of suicidal behavior (27), the relationship between stressful life events, and particularly interpersonal stress and suicidal behavior, has been described in several studies. It was found in a population-based World Health Organization (WHO) World Mental Health Surveys sample of 102,245 adults that traumatic or stressful life events, particularly sexual and interpersonal violence are related to suicidal behavior (28). According to Joiner’s “interpersonal–psychological theory of suicidal behavior”, there are four main predictors of suicidal behavior: thwarted belongingness (feeling alienated/alone), perceived burdensomeness (feeling like being a burden), desire for suicide, and capacity for suicide (20, 29). Serious (lethal or near lethal) suicidal behavior will occur when these constructs co-occur (30). In line with Joiner’s theory, an indirect effect of chronic interpersonal stress on suicidal ideation *via* perceived burdensomeness was also found in adolescent inpatients (31). In a recent study, Stewart and colleagues (32) found in a clinical sample of adolescents that among events categorized as interpersonal loss, physical danger, humiliation, entrapment, and role change/disruption, only interpersonal loss events distinguished suicide attempters from psychiatric controls and suicide ideators, with this effect persisting also when restricting for single attempters and when excluding events following the most recent attempt.

According to Nock’s four-function model on the etiology and maintenance of NSSI (1, 33), self-injury can serve as a seemingly effective method for regulating affective/cognitive experiences and influencing the environment. Thus, factors creating or associated with a predisposition to have problems regulating affective/cognitive state or influencing others in the environment (e.g., physiological hyperarousal as a response to stressful events, unresponsive environment) might increase the risk of the behavior (as well as of other maladaptive coping behaviors). In line with Nock’s model, life events might act both as distal and proximal risk factors for NSSI. As distal risk factors, life events can increase vulnerability to stressors through pathways such as dysregulation of the immune and stress-response systems (34, 35). As proximal risk factors, Kaess and colleagues (24) found that the number of life events, specifically interpersonal events in the past six months predicted the first onset of direct self-injurious behaviors in the following year in a sample of high school students, suggesting that life events might play a critical role in the development of self-injury. On the other hand, findings of Burke and colleagues (36) suggest that this relationship may not be unidirectional: they found in a longitudinal study that engaging in greater NSSI may contribute to the occurrence of interpersonal stressful events among late adolescent girls. In our previous study (37), we compared lifetime prevalence of direct self-injurious behaviors and life event characteristics in high school and vocational school students, a population generally associated with lower socioeconomic status compared to high school students. Vocational school students reported higher prevalence of lifetime self-injury and increased number and severity of life events compared to high school students, but no direct link was found between NSSI and individual life events (37). All these results not only suggest a complex relationship between NSSI and life events but also draw attention to the necessity of including participants from heterogeneous educational settings when studying non-clinical populations.

Despite several results, described above, supporting the role of life events in both suicide and NSSI separately, only a small number of studies have assessed the role of life events in the relationship between the two phenomena. In these studies, the number of stressful life events was found to differentiate between adolescents engaging in suicidal and nonsuicidal self-injury by most (38–40), but not all authors (41). The role of traumatic life events in the relationship between NSSI and suicide was measured in a sample of adolescents by Zetterqvist and colleagues (42): individuals who engaged in both NSSI and attempted suicide differed from those engaging only in NSSI in terms of traumatic life events, that is, adolescents with both NSSI and suicide attempts reported a higher level of adversities and trauma symptoms, and higher rates of interpersonal events when discriminating between interpersonal, non-interpersonal and more longstanding adverse childhood circumstances. The role of interpersonal difficulties in the relationship between NSSI and suicide was also emphasized by Muehlenkamp and colleagues (43): in an outpatient population, adolescents who reported both NSSI and suicide attempts met a higher number of criteria for borderline personality disorder. Among borderline personality disorder features, the severity of confusion about the self and unstable interpersonal relationships were the areas that discriminated most between groups with NSSI only and with NSSI and suicide attempts (43), also highlighting the role of interpersonal difficulties in adolescents presenting both suicidal and nonsuicidal self-injurious behaviors.

The aims of the current study are the following: 1) to explore prevalences of NSSI and suicidal behavior among adolescents in a clinical and non-clinical population, including participants from heterogeneous secondary education settings; 2) to explore the relationship between NSSI and suicidal behavior in the two study groups, and 3) to assess the possible moderating role of stressful life events in the relationship of NSSI and suicidal behavior based on two different aspects: number and type (interpersonal or non-interpersonal) of life events. Moreover, our aim was to screen adolescents with acute suicidal risk and to offer immediate help for those in need by referring them to specialized care services.

Our hypotheses were as follows:

Hypothesis 1. *The number of lifetime NSSI methods is more strongly associated with suicidal behavior in the clinical group compared to the nonclinical group*.
Hypothesis 2. *Higher quantity of life events is associated with an increased number of lifetime NSSI methods in both groups*.
Hypothesis 3. *Interpersonal events have a stronger moderating effect on the relationship between the number of lifetime NSSI methods and suicidal behavior compared to non-interpersonal events and adverse childhood circumstances*.

Furthermore, our aim was to explore if the patterns described in Hypothesis 3 differ between the clinical and non-clinical groups.

## Materials and Methods

Since the methodology of the current study has partly been described previously (44), in the current paper we highlight only the most relevant and additional pieces of information.

### Ethics

The study was approved by the National Scientific and Ethical Committee of Ethics Committees of the Medical Research Council of Hungary (ETT-TUKEB). After being informed of the nature of the study, all participating adolescents and their parents/caregivers gave their oral consent, and all parents/caregivers and adolescents older than 14 years provided written informed consent. In the non-clinical group, parents/caregivers were contacted after getting the consent of school headmasters and head teachers of participating classes.

A code-decode system was used to identify participants at acute suicidal risk based on a structured diagnostic interview (see below); these participants were referred to the specialized health care system.

### Participants and Data Collection

Adolescents between the ages of 13 and 18 were recruited from both clinical and non-clinical settings. The clinical group was recruited from the acute adolescent inpatient department of Vadaskert Child and Adolescent Psychiatric Hospital and Outpatient Clinic, Budapest, Hungary between 25.02.2015 and 09.05.2016. Participants and their parents in the clinical group were contacted and assessed during their time spent in the hospital.

Participants for the non-clinical group were recruited from state-funded high schools, vocational schools and secondary vocational schools in different districts of Budapest, Hungary between 12.09.2015 and 28.04.2017. In this group, parents were contacted at parent–teacher meetings. Adolescents whose parents consented to participate were then contacted and assessed in classroom settings. Overall, 22 classes of children aged 8–11 were contacted. Out of the 185 adolescents with consent from their parent/caregiver, 10 adolescents did not consent to participate; in 14 cases, the parent or the adolescent had their consent withdrawn or adolescents were not available for data collection despite their prior consent (e.g. adolescent was repeatedly absent or has dropped out of school during data collection).

In both groups, exclusion criteria were conditions preventing the completion of self-administered questionnaires (lack of sufficient Hungarian language skills, serious psychiatric states or mental retardation).

### Measurements

Demographic variables, including age and gender, were assessed with a demographic questionnaire developed for the study. This questionnaire was filled out by the parents.

Suicidal behavior was assessed with the Hungarian version of the Mini International Neuropsychiatric Interview Kid (MINI Kid) 2.0 (45–48), a structured diagnostic interview designed for the assessment of major child/adolescent psychiatric disorders. With the suicide module of the interview, both lifetime and current suicidal behavior can be measured. A weighted score belongs to each of the questions of the module, and the total score of the questions answered with a “yes” indicates the level of suicidal risk. Lifetime suicidal behavior is assessed with the following questions: “Have you ever felt so bad that you wished you were dead (score: 1)? Have you ever tried to hurt yourself (score: 2)? Have you ever tried to kill yourself (score: 4)?” Current suicidal behavior is assessed with the questions: “In the past month did you: …wish you were dead (score: 1)? …want to hurt yourself (score: 2)?…think about killing yourself (score: 6)? …think of a way to kill yourself (score: 10)? …attempt suicide (score: 10)?” Scores from 1–5 indicate low suicidal risk, scores from 6–8 indicate moderate risk, and scores of 10+ indicate a high suicidal risk. The interviewer posed the questions of the MINI Kid to the adolescent. The MINI Kid was administered by trained and supervised interviewers.

NSSI was measured with the Deliberate Self-Harm Inventory (DSHI) (49). The DSHI is a behaviorally based, self-administered questionnaire that assesses 16 different methods of NSSI (cutting; burning with cigarette; burning with lighter or match; carving words into skin; carving pictures into skin; severe scratching; biting; rubbing sandpaper on skin; dripping acid on skin; using bleach or oven cleaner to scrub skin; sticking pins, needles or staples into skin; rubbing glass into skin; breaking bones; banging head; punching self; interference with wound healing). The questionnaire offers an “other” option to report NSSI forms not listed in the questionnaire (49).

Life events were measured with the self-administered Life Events List, which was developed for the Saving and Empowering Young Lives in Europe (SEYLE) study based on former literature on life events (24, 50). The questionnaire lists 27 minor and major life events for participants to indicate whether the events were experienced during the six months prior to assessment or not, and offers a 28th item, as “other life event”, to indicate events other than the listed items.

### Statistical Analyses

Data were analyzed using R version 3.6.1. (51). The suicidal behavior variable was calculated based on the number of symptoms reported in the MINI Kid, and this number of suicidal behavior symptoms was weighted with scores of suicide risk severity in MINI Kid. The number of NSSI methods was calculated as the sum of NSSI methods reported in the DSHI (49). Life events were calculated as a sum of 27 life events, excluding item 28 (“Other”). Group was a dichotomous variable (0 = non-clinical, 1 = clinical). Descriptive statistics are reported.

Before estimating the models, the factor structure of the suicidal behavior and self-harm scales were confirmed by factor analysis using the *lavaan* package (52). Since the items in both scales had only two levels, a diagonally weighted least squares (DWLS) estimator was used in the models (53). To guarantee an acceptable level of model fit, five out of the seven fit measures listed below had to be in the acceptable range (see **Table 1** in the **Supplementary Material**). Afterwards, normality of the number of life events, the suicidal behavior weighted sum and the sum of self-harm variables were assessed by separate Shapiro–Wilk tests. Due to normality violations, Wilcoxon tests were used to test differences in suicide, NSSI and life event measures between the clinical and non-clinical groups.

Spearman’s rank correlations between suicide and NSSI with a 95% confidence interval were used to compare the magnitude of the relationship in non-clinical and clinical groups. To estimate whether life events have stronger effect on the NSSI–suicidal behavior relationship in the non-clinical than in the clinical group, we estimated generalized linear models (GLM). The dependent variable was the number of suicidal thoughts and behavior weighted with the suicidal risk presented in the MINI scale. Thus, although the dependent variable is a weighted sum, it still can be treated as a count variable. Consequently, we estimated Poisson regressions, and, in case of overdispersion, we used negative binomial models because ignoring overdispersion can lead to too narrow confidence intervals, inflating the rates of false positives in statistical tests (54). The estimated effect sizes reported in the tables are incident rate ratios (IRR), indicating the percentage change in the dependent variable in response to a one-unit change in the explanatory variable. Similarly to linear regressions, significant interaction effect means an impact over and beyond the main effects.

The distribution of the dependent variable displays an excess number of zeros (indicating the lack of any suicidal behavior for the majority of the participants). It is plausible to assume that distinct processes underlie suicidal behavior and the *lack* of suicidal behavior. In other words, the large number of zeros is not due to “sampling zeros” (meaning that the sampling variation determines the number of zeros, hence an increase in the mean of suicidal behavior would lead to a lower number of zeros), but due to “structural zeros” (55). This structural zero component (the fact that non-suicidality is not the same as an extremely low level of suicidal behavior) requires estimating zero-inflation parameters: otherwise, the model could yield in biased parameter estimates (56).

To take into consideration potential problems concerning both overdispersion and zero-inflation, as well as to check the model diagnostics based on simulated scaled residuals, we used the *glmmTMB* package (57) along with the *DHARMa* package (58). First, we estimated a model with the weighted sum of suicidal behavior as the dependent variable, group membership, number of life events, and number of NSSI methods, and all two- and three-way interactions between them as independent variables.

Regarding life events, beyond the sum of the life events, we created additional explanatory variables to explore the effect of type of stressful life events. Based on the work of Nilsson and colleagues (59) and Zetterqvist and colleagues (42), we sorted life events into three groups: interpersonal, non-interpersonal and adverse childhood circumstances. Seven items were considered interpersonal (such as trouble with parents, breakup with girlfriend/boyfriend), 13 items were considered non-interpersonal (such as failing at an important exam, death of pet), and 8 items were considered adverse childhood circumstances (such as divorce between parents, going to jail) (for all items, see **Table 2** in **Supplementary Material**). Items that could not be matched with any of the items used by Nilsson and colleagues (59) were categorized according to the general classification of life events: events directly linked to an intimate relationship, close friendships, social life and family relationships were considered interpersonal; events linked to academic life, work, financial, personal health and family members’ health were considered as non-interpersonal; and more longstanding, chronic adverse circumstances were considered adverse childhood circumstances (60–62).

## Results

### Sample

Altogether 363 adolescents were involved in the study, 202 of whom (103 girls; 51%) belong to the clinical sample and 161 (80 girls; 50%) of whom belong to the non-clinical sample. For the whole study population, mean age was 15.12 years (SD = 1.31); in the non-clinical population, the mean age was 15.43 years (SD = 1.14); and in the clinical sample, the mean age was 14.87 years (SD = 1.39) (*t*(360) = 4.1, p < .001). From the clinical group, 107 adolescents (53.0%) reported NSSI, while 38 (23.6%) had NSSI from the non-clinical group. Data were missing for 21 participants (for most of the NSSI and stressful life events items), so they were dropped from the database. The final sample consisted of 201 clinical and 141 non-clinical participants.

### Descriptive Statistics and Reliabilities of Study Variables

Results of the confirmatory factor analysis showed excellent fit for both suicidal behavior and NSSI inventories (see **Table 1** in the **Supplementary Material**). Normality was explored by the Shapiro–Wilk test. Results show that the distribution of suicidal behavior (W =.64, p < .001), NSSI (W =.66, p < .001), as well as life events (W =.91, p < .001) violates the normality assumption. Consequently, differences between clinical and non-clinical groups in suicidal behavior, NSSI and life events were tested with Wilcoxon tests. **Table 1** shows the descriptive statistics related to suicidal behavior, NSSI and life events.

**Table 1:** Descriptive statistics of non-clinical and clinical groups.

	Non-clinical group	Clinical group
Suicide—M (SD)	1.45 (4.18)	9.41 (12.03)
Suicide—Mdn	0	3
NSSI – M (SD)	.57 (1.32)	1.84 (2.55)
NSSI – Mdn	0	1
Life events – M (SD)	3.61 (2.49)	5.12 (3.43)
Life events – Mdn	3	5

M, mean, Mdn, median; SD, standard deviation.

### Results of the Statistical Analyses

We found a significant difference related to suicidal behavior W = 7,306, p < .001, NSSI W = 9,652, p < .001, and life events W = 10,410 p < .001 between the non-clinical and clinical groups. The prevalence of suicidal behavior, NSSI and life events was significantly higher in the clinical group than in the non-clinical group of adolescents.

As for the prevalence rate of suicidal behavior (dichotomous variable—is there suicide behavior: yes or no), the presence of any suicidal behavior was a significantly higher (n = 133, 66.2%) in the members of the clinical group than in the non-clinical group (n = 36, 25.5%) (*χ*^2^(1) = 53, *p* < .001). More specifically, significantly higher rate of clinical group (n = 95, 47.3%) engaged in recent suicidal behavior than the non-clinical group (n = 18, 12.8%) (χ^2^(1) = 43, *p* < .001). Additionally, a significantly higher rate of the of members of the clinical group (n = 128, 63.7%) displayed lifetime suicidal behavior compared to the non-clinical group (n = 35, 24.8%) (χ2(1) = 49 p < .001). Moderate suicide risk was found to be significantly higher in the clinical group (n = 26, 12.9%) than in the non-clinical group (n = 3, 2.13%) (χ^2^(1) = 11 p < .001). Finally, a significantly higher rate of the rate of members of the clinical group (n = 66, 32.8%) were at high suicidal risk compared to the non-clinical group (n = 8, 5.67%) (χ^2^(1) = 34 p < .001).

Spearman’s rank correlations with 95% confidence intervals indicate that there is a significant correlation between suicidal behavior and NSSI methods in both groups (non-clinical and clinical). This correlation was significantly stronger in the clinical group (95% CI: [.56,.72]) than in the non-clinical group (95% CI: [.24,.52]). It provides evidence for Hypothesis 1, namely, that NSSI is more strongly associated with suicidal behavior in the clinical group compared to the non-clinical group.

As for the relationship between the number of life events and NSSI methods, the Spearman correlation shows a medium effect size of.38, 95%CI [.28,.46] (63) in the whole sample,.36, CI 95% [.23,.47] in the clinical group, and.31, CI 95% [.16,.46] in the nonclinical group.

After group comparisons, we estimated four regression models. In the following we will highlight the significant effects in the text. We estimated a Poisson GLM with zero-inflation; however, the simulated scaled residuals showed significant overdispersion (ratio of observed vs. simulated residuals: 1.5, p < .001), as well as significant deviation from the assumed distribution (Kolmogorov–Smirnov test D =.17, p < .001) (see **Figure 1** in the **Supplementary Material**). Hence, we re-estimated the model with negative binomial distribution (and zero-inflation), and the diagnostics showed no problems (Kolmogorov–Smirnov test D =.88, p =.34; ratio of observed vs. simulated residuals for dispersion:.88, p =.34; ratio of observed vs. simulated residuals for zero-inflation:.99, p =.93) (see **Figure 2** in the **Supplementary Material**).

In the negative binomial model (**Table 2**), the main effect of NSSI (*χ^2^*(1) = 109.65, p < .001) along with group membership (*χ^2^*(1) = 39.13, p < .001) significantly predicted suicidal behavior; however, the main effect of the number of life events did not explain the dependent variable. The interaction between NSSI and number of negative life events (*χ^2^*(1) = 10.49, p < .01) was significantly associated with suicidality. This indicates that when NSSI is present, higher number of life events is related to higher chance of suicidality over and beyond the main effect of NSSI. However, in this model, it did not differ by groups. Furthermore, neither the effect of life events nor that of the interaction between life events and NSSI differed across groups. This latter finding means that according to this model, compared to the clinical group, stressful life events do not have a stronger effect on the NSSI–suicidality relationship in the non-clinical group.

**Table 2:** Negative binominal regression model: effects of number of life events, group and NSSI on suicidal behavior.

	IRR	Chisq	Df	Pr(> Chisq)
nssi	1.52	109.65	1	<.001***
le	1.12	1.46	1	.23
group	3.75	39.13	1	<.001***
nssi:le	.98	10.49	1	.001**
nssi:group	.91	.88	1	.35
le:group	1.00	0	1	.98
nssi:le:group	1.00	.02	1	.89

le, life event; IRR, incident rate ratio.

**p<.005; *** <.001.

Next, we grouped life events into three categories based on the (59) aforementioned literature and investigated their relationship with suicidality. For non-interpersonal life events, a negative binomial model with zero-inflation showed good fit (Kolmogorov–Smirnov test D =.033, p =.8; ratio of observed vs. simulated residuals for dispersion:.9, p =.4; ratio of observed vs. simulated residuals for zero-inflation: 1, p =.4) (see **Figure 3** in the **Supplementary Material**). Among the predictors, group (*χ^2^*(1) = 40.61, p < .001) and NSSI (*χ^2^*(1) = 137.43, p < .001) were significant. The main effect of life events did not reach significance (*χ^2^*(1) =.05, p =.83), and neither did its interaction with group (*χ^2^*(1) =.69, p =.41), nor the three-way interaction (*χ^2^*(1) =.01, p =.93) (**Table 3**).

**Table 3:** Negative binominal regression model: effects of non-interpersonal life events, group and NSSI on suicidal behavior.

	IRR	Chisq	Df	Pr(> Chisq)
nssi	1.52	137.43	1	<.001***
nipe	.92	.05	1	.83
group	3.26	40.61	1	<.001 ***
nssi:nipe	.98	3.57	1	.06
nssi:group	.84	2.29	1	.13
nipe:group	1.14	.69	1	.41
nssi:nipe:group	1.01	.01	1	.93

Nipe, non-interpersonal life events; IRR, incident rate ratio.

*** <.001.

As for interpersonal life events, the diagnostics were acceptable (Kolmogorov–Smirnov test D =.03, p =.9; ratio of observed vs. simulated residuals for dispersion:.88, p =.3; ratio of observed vs. simulated residuals for zero-inflation: 1, p =.8) (see **Figure 4** in the **Supplementary Material**). Interpersonal life events (IPE) had a significant influence on suicidality (*χ^2^*(1) = 5.77, p =.016) just as group (*χ^2^*(1) = 39.38, p < .05) and NSSI (*χ^2^*(1) = 91.26, p < .001). IPE proved to be a significant moderator of NSSI (*χ^2^*(1) = 19.04, p < .001), indicating that when NSSI is present, higher number of interpersonal life events is related to higher chance of suicidality over and beyond the main effect of NSSI and IPE (**Table 4**).

**Table 4:** Negative binominal regression model: effects of interpersonal life events, group and NSSI on suicidal behavior.

	IRR	Chisq	Df	Pr(> Chisq)
nssi	1.46	91.26	1	<.001***
ipe	1.49	5.77	1	.02*
group	3.49	39.38	1	<.001***
nssi:ipe	.94	19.04	1	<.001***
nssi:group	.96	.38	1	.54
ipe:group	1.02	.01	1	.94
nssi:ipe:group	.9966	0	1	.97

ipe, interpersonal life events; IRR, incident rate ratio.

*p<.05; *** <.001.

Finally, a negative binomial model with zero-inflation for adverse childhood circumstances exhibited a good fit (Kolmogorov–Smirnov test D =.04, p =.7; ratio of observed vs. simulated residuals for dispersion:.9, p =.4; ratio of observed vs. simulated residuals for zero-inflation: 1, p =.4) (see **Figure 5** in the **Supplementary Material**). Neither the main effect of adverse childhood circumstances (*χ^2^*(1) =.34, p =.06) nor the interaction with NSSI (*χ^2^*(1) = 1.21, p =.27) reached significance (**Table 5**).

**Table 5:** Negative binominal regression model: effects of adverse childhood circumstances, group and NSSI on suicidal behavior.

	IRR	Chisq	Df	Pr(> Chisq)
nssi	1.47	157.29	1	<.001***
acc	1.39	.34	1	.56
group	4.69	38.87	1	<.001***
nssi:acc	.94	1.21	1	.27
nssi:group	.90	2.04	1	.15
acc:group	.82	.91	1	.34
nssi:acc:group	1.03	.56	1	.45

acc, adverse childhood circumstances; IRR, incident rate ratio.

*** <.001.

## Discussion

To our knowledge, this study is the first to explore the role of quantity and type of stressful life events in the relationship of NSSI and suicidal behavior in clinical and non-clinical populations of adolescents.

In line with previous findings in the literature, the prevalence of NSSI was significantly higher among psychiatric inpatient adolescents (53.0%) compared to adolescents recruited from heterogeneous educational settings (23.6%). Nevertheless, the lifetime prevalence of NSSI in the non-clinical group was higher in the current sample compared to data on lifetime NSSI prevalence in school samples worldwide (5, 64, 65) and to Hungarian community samples in previous international studies (5, 65), where only high school students were involved. In these previous international comparisons, Hungarian students reported a relatively low prevalence of NSSI with 17.1% according to the SEYLE study (5) and 3.4% for males and 10.3% for females according to the Child & Adolescent Self-harm in Europe (CASE) study (65). Our current results are in line with previous findings, where we found significant differences between high school and vocational school students regarding the prevalence of self-injury in a non-clinical sample of adolescents in Hungary (37). These results call attention to the necessity of including adolescents from various educational settings in both research and prevention projects.

Regarding suicidal behavior, although both lifetime and current suicidal behavior were significantly higher in the clinical group, alarmingly high rates of suicidal behavior were reported in the non-clinical group, as well: a quarter of adolescents reported some level of suicidal behavior (suicidal ideation or attempts) during their lifetime, and more than one-tenth of them did so in the last month prior to assessment. More specifically, moderate suicidal risk was assessed in 2.13% of the adolescents, and 5.67% of adolescents were at high suicidal risk at the time of the assessment. Screening for these adolescents and referring them to the specialized health care system was an important aim of our study.

The high prevalence of both suicidal and nonsuicidal self-injury in the non-clinical group are especially alarming considering possible bias of data collection. Adolescents who were unavailable for inclusion in the study might be at an even more elevated risk: school staff and parents who were unresponsive or refused participation might have a decreased level of involvement, and/or a general rejective attitude towards mental health prevention. Additionally, frequent absence or dropout from school might also indicate the presence of an increased number of risk factors. Thus, the prevalence of self-injurious behaviors in this population might be even higher than reported.

Regarding the relationship between NSSI and suicidal behavior, NSSI proved to be associated with suicide in both groups, and this association was significantly stronger in the clinical than in the non-clinical group. These results are in line with studies that describe NSSI and suicidal behavior as frequently overlapping (11, 14, 15). Previous findings in the literature support both NSSI being a risk factor for suicidal behavior (11) and the presence of third variables behind both NSSI and suicidal behavior (16, 17, 21), and do not discard the idea that individuals who engage in suicidal behavior are at increased risk for NSSI (17). Group differences in particular raise the possible role of mental disorders as mediating variables between the two phenomena, or as third variables behind both NSSI and suicidal behavior. Although individuals who engage in NSSI often report anti-suicidal functions of NSSI (66, 67), according to Kiekens and colleagues, NSSI increases, rather than decreases, the risk of turning suicidal ideation and urges into acts of suicidal behavior (4), underlining the importance of prevention and intervention for those who engage in NSSI.

Another possible third variable can be the presence of stressful life events. In line with previous findings (24, 25), in the present study, the number of life events experienced was associated with NSSI. According to our results, a higher number of life events was correlated with an increased number of NSSI methods in both groups, but had no main effect on suicidal behavior in either of the groups. Nevertheless, for those adolescents who engaged in NSSI, the number of stressful life events proved to be an important factor in also engaging in suicidal behavior. Although when life events were not considered, we found group differences for the NSSI–suicidality association, when we controlled for life events, this relationship was no longer significant. Hence, experiencing life events may be a potential (third) factor behind group differences in both NSSI and suicidal behavior.

(68) When investigating life events based on their type (interpersonal or non-interpersonal events or adverse childhood circumstances), only interpersonal events proved to be associated with both suicidal behavior and had a moderating effect on the NSSI–suicidality relationship. This is in line with previous findings of Zetterquist and colleagues (42) on the role of interpersonal events. The association between interpersonal conflicts and NSSI suggests that these events might be highly triggering for adolescents vulnerable to NSSI, and highlight the role of possible intra- and interpersonal factors contributing to the increased risk of both interpersonal conflicts and NSSI [e.g. difficulties with emotion regulation, an environment that is unresponsive to the adolescent’s needs (1, 10)]. According to Burke and colleagues (36), who found in late adolescent girls that the frequency of lifetime and past year NSSI predicted the occurrence of interpersonal stressful life events at follow-up beyond the effects of initial depressive symptoms, the idea that engagement in NSSI might also contribute to interpersonal life events, should also not be discarded. Besides these life events occurring as specific interpersonal consequences of NSSI (36) (e.g. related to the stigma associated with the behavior), it is also possible that NSSI as a maladaptive mechanism for communicating and coping with interpersonal difficulties might prevent the individual from solving interpersonal conflicts in adaptive ways, thus contributing to interpersonal life events (e.g. serious argument, break-up). According to Joiner’s “interpersonal–psychological theory of suicidal behavior”, serious suicidal behavior will occur when the main predictors of suicidal behavior—thwarted belongingness, perceived budernsomeness, desire for suicide and capacity for suicide (20, 29) co-occur (30). In line with this model, those with co-occurring interpersonal life events (potentially contributing to thwarted belongingness, perceived budernsomeness) and NSSI (potentially contributing to increased capacity for suicide) can be at high risk for suicidal behavior. Our results support the findings of Zetterquist and colleagues (42) on the role of interpersonal events and Muehlenkamp and colleagues on the interpersonal features and functions of NSSI (69), who conclude that besides emotion regulation, treatments should also focus on strengthening interpersonal bonds. When interpreting our results on this issue, it should be considered that the instrument used in the current study focused primarily on stressful, but not on traumatic life events specifically; moreover, only life events in the six months prior to assessment were explored. Thus—although results are controversial about how some adverse childhood events or traumas, for example, childhood sexual abuse contributing to the etiology of NSSI (70), these results do not necessarily conflict with general findings in both clinical and non-clinical samples (71) on the role of several forms of adverse childhood circumstances and maltreatment related to engaging in self-injurious behaviors.

Although clinical and non-clinical groups differed significantly not only in the prevalence of NSSI and suicidal behavior but also in the number of life events reported, the patterns described above of the effects of life events on the NSSI–suicidality relationship did not differ in the two groups. This result can indicate that these patterns might be associated with the aforementioned functions of NSSI being frequent in both clinical and non-clinical populations. When interpreting our results, it should also be considered that some of the life event labels (e.g. new family member, minor violation of law) can cover a wide range of personal experiences. Thus, it is possible that similar answers on the life event list refer to different severities of experiences for participants in the two groups.

### Limitations and Future Directions

Our results need to be interpreted with the consideration of the limitations of our study. The cross-sectional nature of our data does not provide information about causality. Despite our efforts to minimize these effects with constant supervision and providing help in understanding the questions, possible bias due to the self-administered questionnaires should also be considered. It is a possible direction for future research to further develop different facets of the life event inventory.

Furthermore, exploring the role of sociodemographic factors (e.g. differences related to gender, socioeconomic status) and the role psychiatric disorders was out of the cope of this study; the possible effects of these phenomena should be further explored in future research.

## Conclusions

The high prevalence of NSSI and suicidal behavior in both clinical and non-clinical groups indicates urgent need for prevention and intervention programs not only in clinical settings, but also in secondary education schools, including both vocational and high school education. Our results highlight that prevention and intervention of NSSI is especially important, since the behavior frequently co-occurs with suicidal behavior in both the clinical and non-clinical population. Moreover, targeted prevention should consider focusing on adolescents who experience a high number of life events, since a higher number of these events might co-occur with an increased number of NSSI methods—which, according to several studies, might be a key indicator for NSSI severity (72, 73)—and with engaging in both NSSI and suicidal behavior. Interpersonal life events, such as trouble with parents, a serious argument with a close friend or teacher, and/or a breakup with a partner, are associated with suicidal behavior and moderate the relationship between NSSI and suicidal behavior. To support the prevention and treatment of NSSI and suicidal behavior, the presence of stressful life events in the life of adolescents requires special attention.

## Data Availability Statement

The raw data supporting the conclusions of this article will be made available by the authors, without undue reservation, to any qualified researcher.

## Ethics Statement

The studies involving human participants were reviewed and approved by National Scientific and Ethical Committee of Ethics Committees of the Medical Research Council of Hungary (ETT-TUKEB). Written informed consent to participate in this study was provided by the participants’ legal guardian/next of kin.

## Author Contributions

LH participated in the design of the study, performed the literature search, participated in the data collection and analyses, and drafted the manuscript. DG participated in the data collection and analyses and drafted the manuscript. DK performed the data analyses and drafted the manuscript. GM participated in the design of the study, participated in data collection and analysis, and drafted the manuscript. DS participated in the data collection and analysis and drafted the manuscript. JB was the principal investigator of the study, led the design of the study, coordinated the steps of the data collection and data analyses and drafted the manuscript. All authors contributed to manuscript revision, read and approved the submitted version.

## Funding

Supported by the ÚNKP-19-3 New National Excellence Program of the Ministry for Innovation and Technology. JB was supported by the János Bolyai Research Scholarship of the Hungarian Academy of Sciences. This work was supported by the OTKA K108336 grant.

## Conflict of Interest

The authors declare that the research was conducted in the absence of any commercial or financial relationships that could be construed as a potential conflict of interest.
