# Supplementary material for: Nonsuicidal Self-Injury and Suicide: The Role of Life Events in Clinical and Non-Clinical Populations of Adolescents
Source: Front Psychiatry. 2020 May 6;11:370. doi: 10.3389/fpsyt.2020.00370 (PMC7218062; doi:10.3389/fpsyt.2020.00370)
Supplement: Supplementary file 1 [file DataSheet_1.docx]

Nonsuicidal self-injury and suicide: the role of life events in clinical and non-clinical populations of adolescents

Lili Olga Horváth^1,2*^, Dóra Győri^1,2^, Dániel Komáromy^2,3^, Gergely Mészáros^4,5^, Dóra Szentiványi^1,2,6^, Judit Balázs^1,7^

^1^Doctoral School of Psychology, Eötvös Loránd University, Budapest, Hungary

^2^Institute of Psychology, Eötvös Loránd University, Budapest, Hungary

^3^Faculty of Behavioural and Movement Sciences, Vrije Universiteit Amsterdam, The Netherlands

^4^Semmelweis University, Mental Health Sciences School of Ph.D., Budapest, Hungary

^5^Vadaskert Child Psychiatry Hospital and Outpatient Clinic, Budapest, Hungary

^6^Pedagogical Services, Budapest, Hungary

^7^Bjørknes University College, Oslo, Norway

*** Correspondence:**Lili Olga Horváth
horvath.lili@ppk.elte.hu

# Supplementary Material

Table 1. Threshold values and fit indices for suicidal inventory and NSSI inventory (factor analytic models)

| **Threshold values** | | | | | | | |
| --- | --- | --- | --- | --- | --- | --- | --- |
| rmsea | cfi | tli | rni | ifi | srmr | agfi |  |
| 0.08 | 0.90 | 0.90 | 0.90 | 0.90 | 0.08 | 0.90 |  |
| **Suicidal behaviour inventory** | | | | | | | |
| rmsea | cfi | tli | rni | ifi | srmr | agfi |  |
| 0.04 | 0.994 | 0.992 | 0.994 | 0.994 | 0.073 | 0.993 |  |
| **NSSI inventory** | | | | | | | |
| rmsea | cfi | tli | rni | ifi | srmr | agfi |  |
| 0 | 1 | 1.007 | 1.006 | 1.006 | 0.1 | 0.914 |  |

Table 2. Categorization of the life event items by type

| Life event | Type  (nIPE/IPE/ACC) |
| --- | --- |
| Serious argument with a teacher | IPE |
| Trouble with parents | IPE |
| Theft of personal belongings | IPE |
| Sex problems | IPE |
| Serious argument with a close friend | IPE |
| Breakup with girlfriend/boyfriend | IPE |
| Parent stopped or started work | nIPE |
| Increased workload at school | nIPE |
| Lower grades than expected | nIPE |
| Marriage of emotionally close sibling | nIPE |
| Appearing for an exam, interview | nIPE |
| New family member | nIPE |
| Change in financial status of parents | nIPE |
| Change of school | nIPE |
| Minor violation of law | nIPE |
| Failed important course/exam | nIPE |
| Death of pet | nIPE |
| Death of close friend | nIPE |
| Death of close family member | nIPE |
| Change in family member’s health | ACC |
| Alcohol/drug use in family | ACC |
| Trouble with bullies | ACC |
| Parents became unemployed | ACC |
| Jail term | ACC |
| Major personal injury or illness | ACC |
| Divorce between parents | ACC |
| Pregnancy | ACC |

IPE =interpersonal events; nIPE = non-interpersonal events; ACC = adverse childhood circumstances

Figure 1. Diagnostics of the Poisson model

| 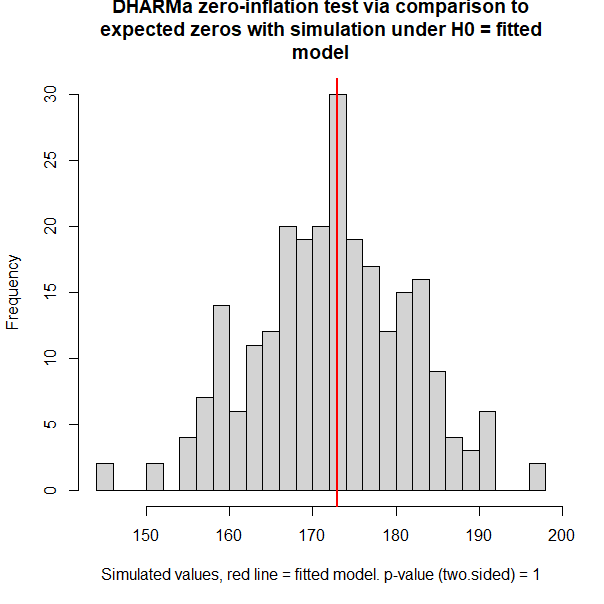 | 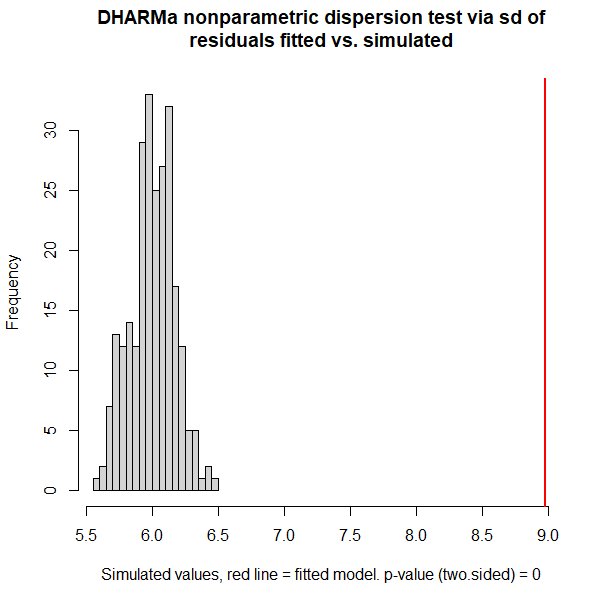 |
| --- | --- |
| 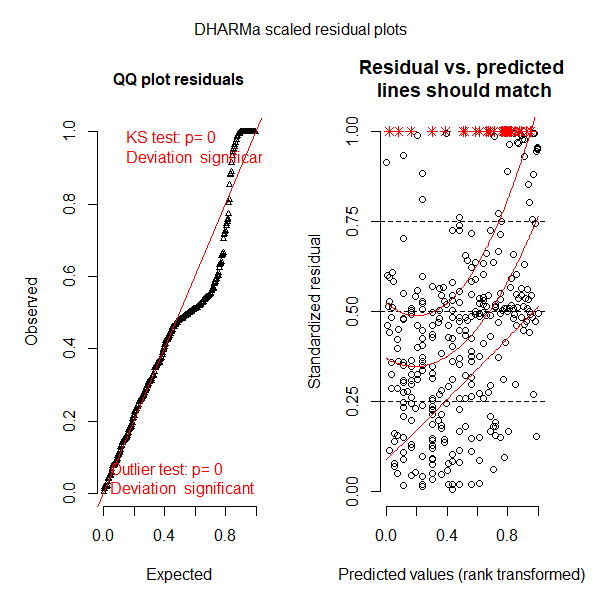 |  |

Figure 2. Diagnostics of the negative binomial model

| 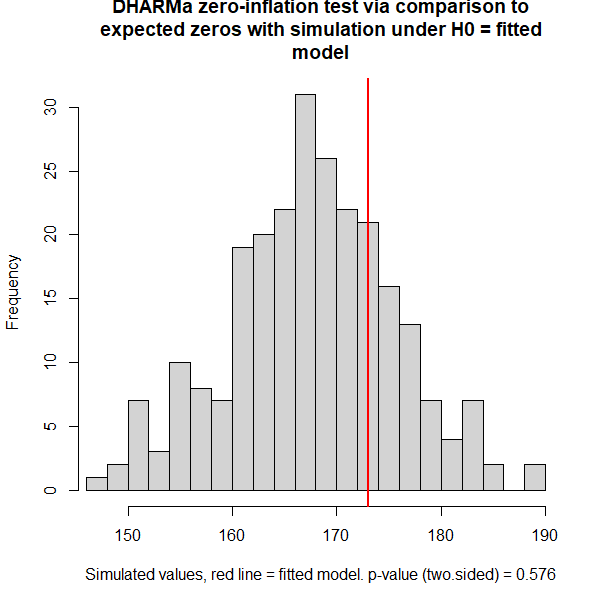 | 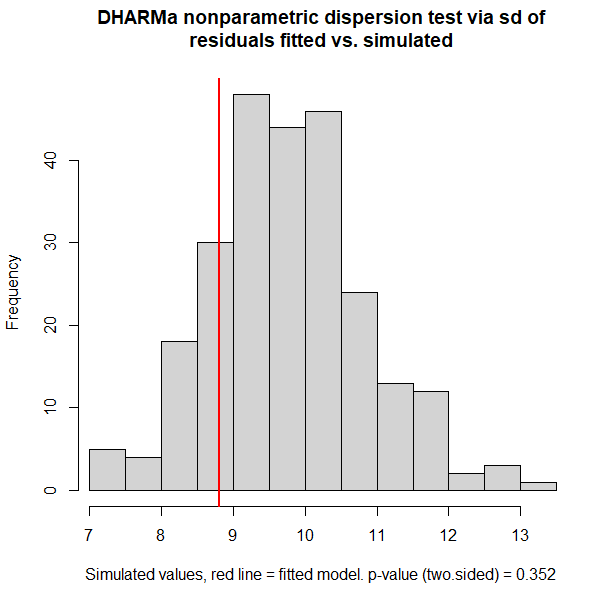 |
| --- | --- |
| 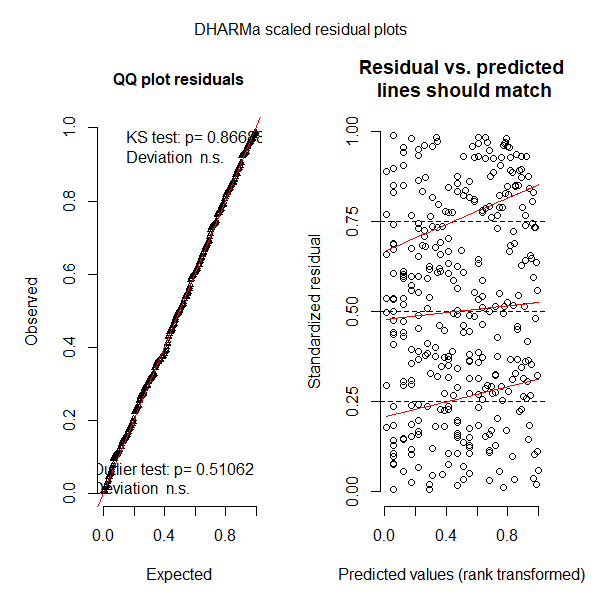 |  |

Figure 3. Diagnostic of the negative binominal model for Non-interpersonal events

| 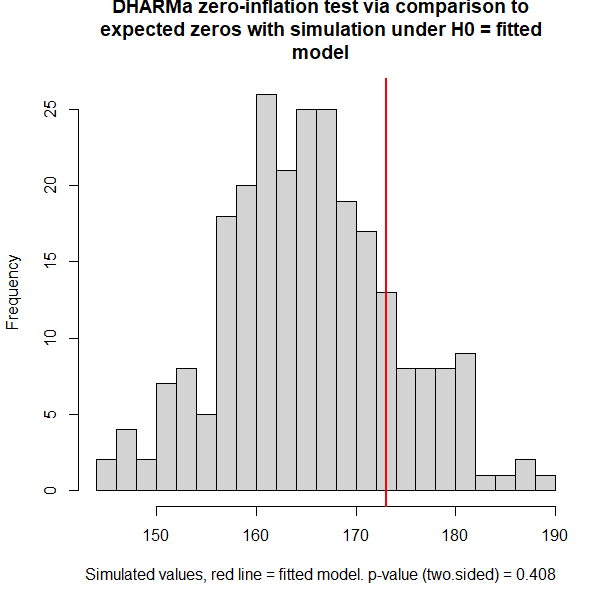 | 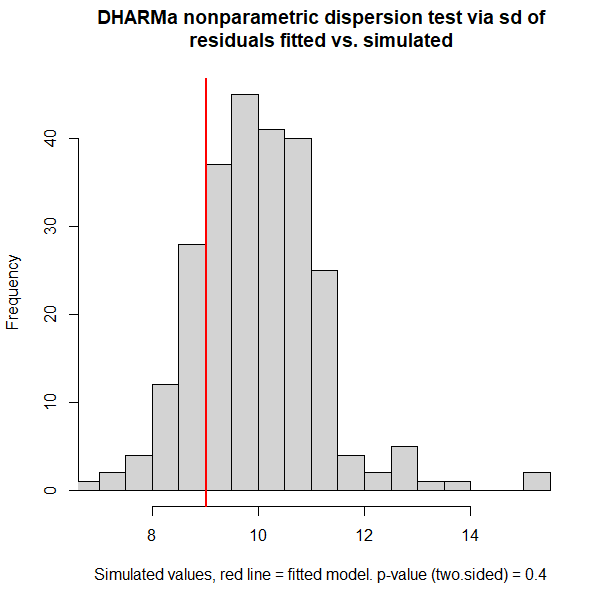 |
| --- | --- |
| 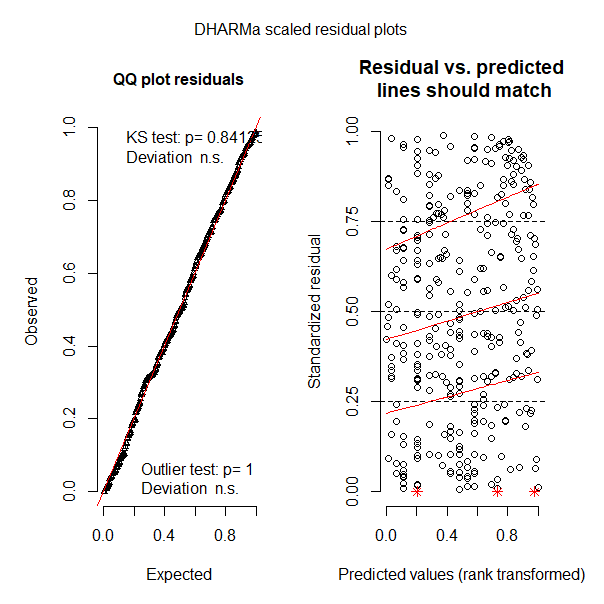 |  |

Figure 4. Diagnostic of the negative binominal model for Interpersonal events

| 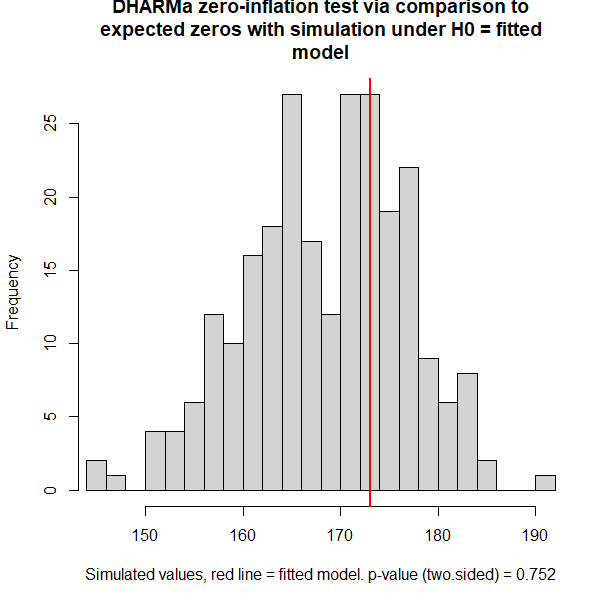 | 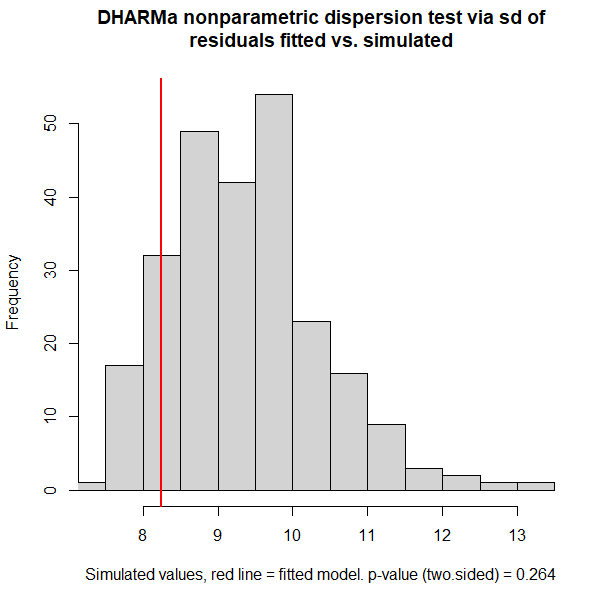 |
| --- | --- |
| 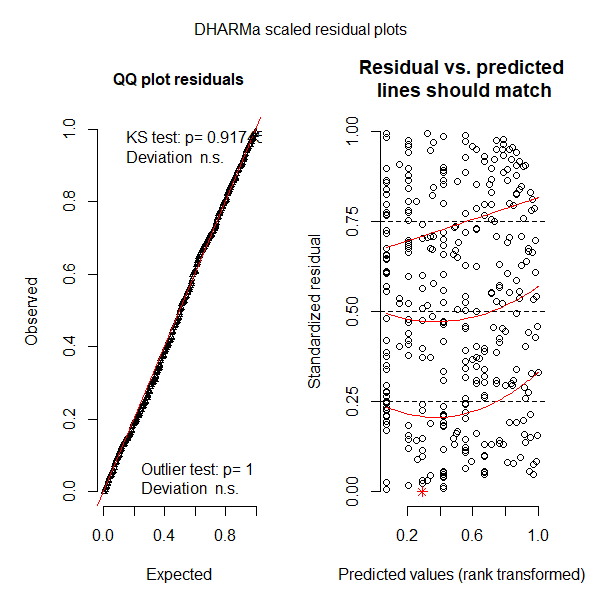 |  |

Figure 5. Diagnostic of the negative binominal model for Adverse Childhood Circumstances

| 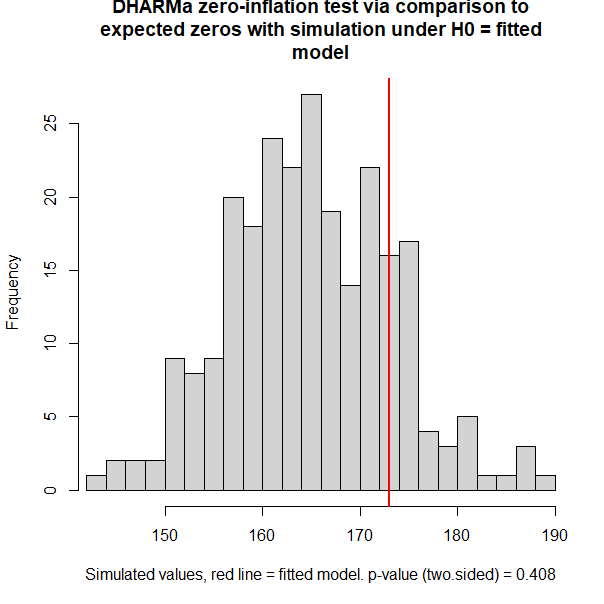 | 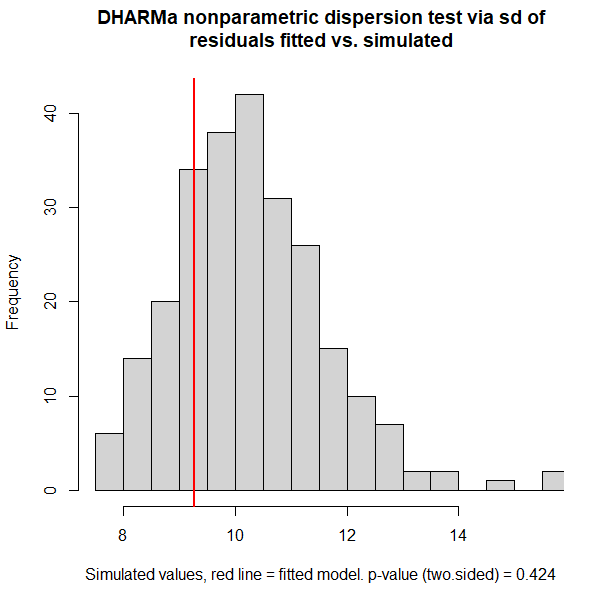 |
| --- | --- |
| 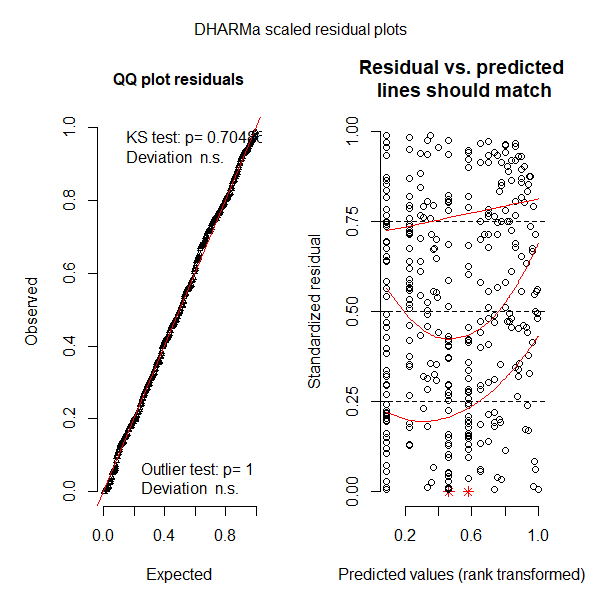 |  |
